# Male Partner Involvement in Birth Preparedness, Complication Readiness and Obstetric Emergencies in Central Rural India: A Cross-Sectional Study

**DOI:** 10.7759/cureus.60148

**Published:** 2024-05-12

**Authors:** Shubhangi Arohee, Amey A Dhatrak, R Naveen Shyam Sundar

**Affiliations:** 1 Community Medicine, Mahatma Gandhi Institute of Medical Sciences (MGIMS), Sevagram, IND

**Keywords:** nursing mother, family relationship, maternal-child health, birth preparedness and complication readiness, male partner involvement

## Abstract

Background and objectives: Childbirth is mainly thought to be a woman’s concern, and mortality can be prevented by making a birth plan constituting birth preparedness and complication readiness with the entire family as one unit. Indian National Plans aim to increase male involvement, but the policies lack directions and monitoring systems; hence, it becomes important to address this issue.

Methodology: A cross-sectional study conducted in a rural hospital and a community-based setup included 350 male participants, consisting of new fathers or expecting fathers, who were interviewed with the help of a questionnaire.

Results: Only 28.29% of male participants were well involved in the process of birth preparedness and complication readiness. 83% of the husbands accompanied their respective wives during ANC visits (mean number of visits: 5.76). 33% of males were aware of various danger signs and complications related to pregnancy. The males with better education (p-value < 0.005) and economic status (p-value < 0.0001) had better birth preparedness. Several variables in the study were positively correlated with the amount of money saved.

Interpretation and conclusion: Male involvement during pregnancy significantly impacts maternal and child health outcomes. However, this study highlights a lack of awareness and involvement among males. We strongly recommend enhancing existing maternal and child health (MCH) programs to include components focused on male partner engagement in birth preparedness, complication readiness, and obstetric emergencies.

## Introduction

Worldwide, it is estimated that 2.8 million pregnant women and newborns pass away each year from preventable causes of delivery problems or one every 11 seconds. In 2017, problems during pregnancy and childbirth claimed the lives of more than 800 women globally each day [1]. The maternal mortality ratio in India has declined to 103 in 2017-19 from 113 in 2016-18 [2]. In 2019-20, the neonatal mortality rate was 17.6, and the infant mortality rate was 23.7 in the rural population of Maharashtra, where this study was conducted [3]. India's National Rural Health Mission promotes expert birth and emergency obstetric care with financial incentives like free transportation [4]. India's national plans aim to increase male engagement in women's health programs but lack clear policy directions and monitoring systems [5].

Deaths due to pregnancy, childbirth, and postpartum problems can be prevented by making a birth plan that constitutes birth preparedness and complication readiness (BPCR) for pregnant women, their partners, and their family members [6]. The three delays at the time of delivery, namely 'delays in seeking' and 'receiving' care, are major contributors to maternal mortality. To tackle all these delays, it is very important to have a birth plan ready beforehand. This is where BPCR gains its importance. BPCR refers to a plan organized during pregnancy for preparations for normal delivery and complications if the situation arises [7]. 

Male participation in prenatal care has been linked to better results for mother and child [8]. Active partner participation in maternal care can significantly reduce MMR, with male involvement accelerating development and solidifying earlier achievements. [9]. Education and training the couple together about complications and emergencies can promote a healthy pregnancy and a healthy baby. This study was conducted to understand the involvement of male partners in birth preparedness and analyze male birth preparedness and complications awareness, identifying methods to increase awareness and identifying knowledge gaps to provide community interventions in the future.

Aims and objectives

The primary objective of this study is to assess the involvement of male partners in birth preparedness and complication readiness during the antenatal and postnatal periods.

The secondary objectives of this study aim to assess the accessibility to healthcare services in rural populations during pregnancy (ANC/PNC period) and the knowledge of the husband regarding danger signs during pregnancy.

## Materials and methods

A cross-sectional study design was employed to achieve the research objectives during the study's duration, spanning from July to October 2022. This research effort unfolded in two distinct settings. Firstly, it encompassed a hospital-based setting, taking place in a rural tertiary care hospital located in Wardha, Maharashtra, where consecutive sampling was utilized. Secondly, a community-based setup was also included, with villages selected from the field practice area of the Medical College, specifically the Mahatma Gandhi Institute of Medical Sciences (MGIMS), Sewagram. The villages were chosen using simple random sampling.

The study's participants consisted of husbands whose wives were either in the last trimester of pregnancy or had given birth within the last 20 weeks. The rationale behind this selection criteria was twofold: to avoid recall biases and to engage couples when they are at a heightened state of awareness, especially during the last trimester, given the increased risk of emergencies and complications such as pain, bleeding, pregnancy-induced hypertension, and gestational diabetes mellitus.

The sample size was calculated based on the findings of a recent study from India, indicating that approximately 70% of husbands participated in birth preparedness and complication readiness [9]. This calculation yielded a sample size of 323 to detect differences at a 95% confidence interval, with a 10% precision level and a power of 80%, assuming a design effect of 1. To account for a 5% non-responsive rate, the estimated sample size for the study was determined to be 350. Consequently, a total of 350 males were interviewed using a structured questionnaire designed for the study.

The inclusion criteria stipulated that the participants must be husbands of pregnant females in their last trimester or fathers of infants under 20 weeks old. Additionally, couples who did not reside together were excluded from the study. Ethical considerations were of paramount importance, and the study received approval from the Institutional Ethics Committee (IEC). Fully informed consent was obtained from each participant before their enrolment in the study, and strict measures were implemented to uphold privacy and confidentiality throughout the data collection process.

Data collection

Data was collected from both hospital and community settings, each accounting for 50% of the total sample size. The hospital chosen for the study was a tertiary care facility located in Wardha, serving a patient catchment area of approximately 90 villages within a 50-km radius. Within the hospital setting, participants were requested to complete the questionnaire while in the ward and Outpatient Department (OPD) of the Maternal and Child Health (MCH) wing. The active participation of hospital subjects was solicited to ensure the questionnaire's thorough completion.

In the community-based setting, a total of 10 villages were randomly selected from the field practice area of MGIMS, Sewagram. A comprehensive list of pregnant couples and new parents who met the defined study participant criteria was compiled with the assistance of accredited social health activists (ASHA workers) from the respective villages. Subsequently, home visits were conducted to administer the questionnaire to the identified participants.

The structured questionnaire utilized in this study was adapted from a previously validated questionnaire employed in studies focusing on male involvement in birth preparedness and complication readiness [10-13]. Our final questionnaire (Annexure I) was done based on all four different studies, and pilot testing was undertaken within the mother and child health (MCH) wing of the Medical College, involving 15 respondents for each category, resulting in a total of 30 interviews. After pilot testing, expert and peer reviews were conducted, leading to necessary modifications and refinements. The final data collection was done through structured interviews with the participants both in the hospital and in the community. It was assured that each participant gets enough time to answer the question, and we don't have any time limits for the same. The questionnaire administration and the data collection were done by the authors themselves, and no specific data collectors were appointed, ensuring robustness during data collection.

Data analysis

The collected data was meticulously examined, cleaned, and organized using data analysis software, including Excel, Epi Info, and R. In line with the study's objectives, several key areas were scrutinized. Specifically, male preparedness was meticulously assessed based on a scoring system (Appendix) developed for this study. These scores were categorized into three distinct levels: 'not involved, ''moderately involved,' and 'well-involved,' offering a nuanced perspective on male participation in birth preparedness.

Socio-demographic factors, including age, education, occupation, and economic status, were subjected to rigorous analysis to uncover potential correlations with levels of preparedness. The influence of previous complications in pregnancy on birth preparedness was meticulously examined, allowing for insights into whether prior experiences influenced subsequent readiness for childbirth. Furthermore, the study delved into the knowledge base of participants regarding danger signs in pregnancy, shedding light on areas that may require targeted educational interventions.

## Results

The research outcomes are poised to illuminate the status of male involvement in birth preparedness and complication readiness in the rural Indian context. Initial insights reveal that a substantial proportion of the study participants, specifically 86% (n=303), reported having completed their education up to the 12th grade. This observation suggests a relatively high level of educational attainment among the respondents. Additionally, the total participants of the study were 350 (N) individuals, reflecting a robust sample size for the investigation. These findings, as presented in Tables 1, 2, underscore the significance of examining the role of male education and socio-economic status, particularly concerning birth preparedness and complication readiness within rural communities. Further analysis of the data from this study promises to offer valuable insights into strategies for enhancing male involvement in maternal healthcare initiatives.

**Table 1:** Male involvement in birth preparedness and complication readiness

Total score	Number of males (%)	Involvement
2 to 4	52 (14.86)	Low involvement
5 to 7	199 (56.86)	Moderate involvement
8 to 10	99(28.29)	Well involved

**Table 2:** Male involvement in birth preparedness and complication readiness as per socio-demographic characteristics *chi-square test of significance, p-value < 0.05 is significant

Categories	Sub-categories	Number of individuals with total score			p-value
		2-4	5-7	8-10	
Male education	Below class 10	4	5	1	0.0059
	10th	5	26	6	
	12th	24	56	28	
	Graduate and postgraduate	19	112	64	
Ration Card	Marginalized	16	38	8	0.00012
	BPL	15	54	16	
	APL	21	107	75	
Type of Family	Nuclear	11	67	26	0.267
	Joint	17	49	33	
	Three generation	24	83	40	

**Figure 1:**
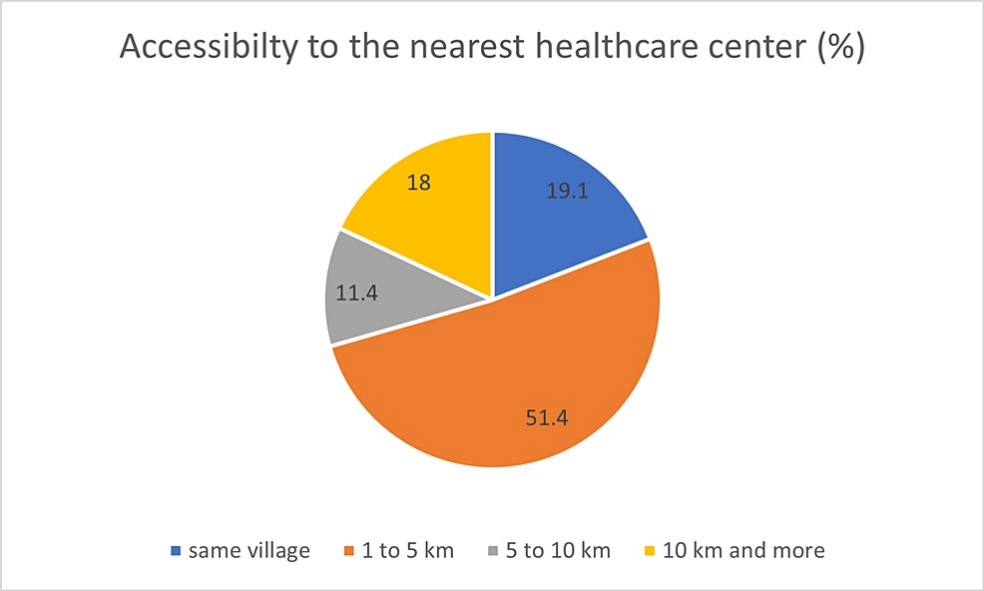
Distribution of population according to the distance traveled by them to reach the nearest healthcare center

Continuing in the spirit of preparedness, the study probed the participants' awareness regarding logistical arrangements for potential childbirth complications and emergencies. Impressively, 92% (n=322) of the male respondents were able to identify a mode of transportation to facilitate rapid access to healthcare services in case of emergencies. This finding underscores the proactive approach adopted by these husbands in readiness for any exigencies (Figure 1).

Similarly, the study revealed that an overwhelming majority of participants, constituting 95.4% (n=334), were able to discern the birthplace or hospital where they intended to seek medical care in the event of an emergency. This proactive identification of healthcare facilities further bolsters the notion that these couples are earnestly invested in ensuring a safe and healthy childbirth experience. 

Furthermore, the research findings indicated that 91% (n=319) of the male participants had already made a definitive decision regarding the birth attendant. This decision-making process is critical to ensuring that skilled healthcare professionals are on hand to provide appropriate care and guidance during childbirth, thereby reducing the risk of complications and mortality.

Intriguingly, a significant 77% (n=269) of the male participants indicated that they had actively saved money in anticipation of childbirth and potential emergencies. The cumulative average of these savings was reported to be approximately Rs 21,769.35, highlighting the financial commitment undertaken by these husbands to safeguard the health and well-being of their wives and newborns. However, the study also unearthed areas where preparedness was less pronounced. Notably, only 37% (n=130) of the male respondents reported having identified a potential blood donor who could be called upon in the event of a critical medical situation.

Beyond financial and logistical preparedness, the study delved into the dynamics of male participation in antenatal care (ANC) visits. Approximately 83% (n=291) of the male respondents reported accompanying their wives to ANC visits. In contrast, the study exposed a less encouraging facet of male involvement. Specifically, only 35% (n=123) of the participants reported having engaged in discussions with the village Accredited Social Health Activist (ASHA) regarding pregnancy-related issues. This observation hints at a potential gap in the communication and engagement strategies employed by healthcare workers in rural areas.

**Figure 2:**
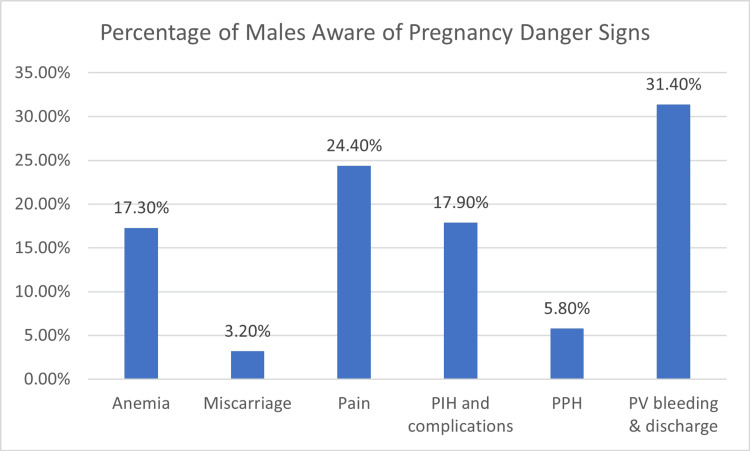
Percentage of males aware of pregnancy danger signs

Furthermore, the study assessed the level of knowledge among the participants regarding danger signs in pregnancy. Alarmingly, approximately 33% (n=116) of the male respondents admitted to being unaware of any danger signs associated with pregnancy (Figure 2). This finding is particularly concerning, as it underscores a critical knowledge gap that could hinder timely and appropriate responses in the face of pregnancy complications.

Also, Table 3 presents correlations between various factors related to maternal health and pregnancy, along with their respective Pearson correlation coefficients (r values) and p values. We used correlation analysis to understand how different factors are related to each other so that we could identify the factors to be recommended for better male participation during pregnancy and for guiding efforts toward a better maternal outcome. Among the correlations listed, three are statistically significant at the 0.05 level. There is a negative correlation between complications in previous pregnancies and the amount of money saved (r = -0.105, p = 0.049), indicating that individuals with a history of complications tend to save less money. There is a positive correlation between the identification of a birth kit and awareness about danger signs during pregnancy (r = 0.13, p = 0.0087), indicating that individuals who are more aware of danger signs are also more likely to identify and utilize a birth kit. These findings shed light on important associations between socio-economic factors, health awareness, and preparedness for pregnancy and childbirth, emphasizing the importance of correlation analysis in understanding the multifactor determinants of maternal health in rural India.

**Table 3:** Correlation between variables along with their r-value and p-value *the p-value <0.05 is considered significant, t-statistics for correlation have been applied

Variables	R-value	P-value
Distance from the health facility and amount of money saved	0.06	0.214
Complication in previous pregnancy and amount of money saved	-0.105	0.049
Identification of birth kit and awareness about danger signs	0.13	0.0087
Ration card and distance	0.09	0.082
Complication in previous pregnancy and knowledge of danger signs	0.10	0.048
Health facility chosen and money saved	0.13	0.009
Ration card and health facility chosen	0.10	0.050

## Discussion

Male involvement in birth preparedness is crucial due to demographic shifts and family structure changes. Our study found that 56.8% of male participants (n=199) were prepared for 5-7 components on the BPCR scale, while only 50% (n=175) had a birth kit for emergencies. Only 77% (n=269) saved money for delivery or emergencies, and only 37% (n = 129) identified a blood donor. However, 92% (n=322) identified transport, 81% (n=283) planned leave for delivery, and 95.5% (n=334) identified the place of birth or healthcare facility. The study found that 70.5% (n=247) of participants lived within a five-kilometer radius of healthcare facilities, while 18% (n=63) had to travel 10 km or more. Man's knowledge of danger signs was divided into themes, with 31% (n=109) focusing on vaginal bleeding and discharge, while others focused on abdominal pain and pregnancy-induced hypertension (PIH) complications.

When comparing these findings with a study done in 2018 from Bangladesh by Sajia Islam et al, it was found that in their study, 75% of couples had identified a place of birth, compared to 95.5% in our study (n=334). Similarly, they found that 72% had identified a birth attendant, while in our study, 91% had done so (n=318). In terms of arranging for transport, their study reported 13%, contrasting with 92% in our study (n=322). Furthermore, they found that 38% had saved money for emergencies, whereas in our study, this figure was 77% (n=269). Finally, in their study, only 8% had identified blood donors, while in our study, it was 37% (n=129). These comparisons underscore the variations in birth preparedness and complication readiness between the rural Indian context and Bangladesh, highlighting potential areas for intervention and improvement in both settings. 

Our study found that 77% (n=269) of the population in the study had saved money for emergencies, compared to 81% and 42.8% in Zambia [14] and Malawi [15], respectively. This variability is influenced by education status, socio-cultural factors, and rural-based settings, where people often have social security, such as health insurance, compared to urban areas.

Only 50% (n=175) of men identified a birth kit, with awareness of pregnancy danger signs significantly impacting identification. Tanzania and Ethiopia have similar rates, but Ethiopia is far behind in the same aspect [16,17].

In this study, it was observed that a significant proportion, 81.1% (n=283) of the study population, had planned to take leave around the time of delivery. This marked contrasted with findings from a 2016 study conducted in Uttar Pradesh, India, where only 19.7% of men had planned to take leave [12]. Several factors could account for this shift over time. It is plausible that increased awareness and consideration regarding birth preparedness and complication readiness (BPCR) have evolved with time. Additionally, various state-sponsored schemes and the emergence of 'Paternity Leave' may have played a role in encouraging male partners to take time off during their wives' deliveries. The average duration of leave planned by husbands was approximately 10 days, with factors like occupational circumstances, such as farmers and shopkeepers tending to their businesses or professionals working in the same hospital where their wives were registered, influencing their decisions. On the other hand, daily wage laborers were concerned about financial losses and often opted to continue working, with relatives accompanying their wives to the hospital. Normalizing paternity leave and providing paid leave, especially in the unorganized sector, could potentially further increase male participation during delivery and the post-pregnancy period.

Birth planning and preparedness entail financial considerations as child-rearing expenses increase. Comparing this study with previous research, it becomes evident that awareness and education have contributed to an increase in the proportion of men saving money for emergencies. In this study, 75% (n=262) of the population saved money for emergencies, a significant improvement over a 2011 study in Rural Uganda where only 1/3rd of male participants saved money, and a 2018 study in Karnataka where 58% did [12,14]. However, it's important to acknowledge that inflation and a higher standard of living have also driven up costs, which may have played a role in this increase. Among the remaining 1/4th of the population that did not save, some were simply unaware of potential complications and emergencies, emphasizing the need for increased education on these matters. Hospital employees, who expected the hospital to cover all expenses, were also less inclined to save. Bridging knowledge gaps and providing information on the potential costs associated with complications and emergencies could help improve birth planning and encourage financial preparedness. Moreover, a concerning inverse relationship was found between complications in previous pregnancies and money saved, highlighting the vulnerability of poorly educated couples in rural areas to emergencies. Addressing this gap in healthcare accessibility and knowledge is paramount to ensuring safe deliveries.

In terms of geographical access to healthcare facilities, 29.4%(n=102) of the population lived more than 5 km away, raising concerns about emergencies. This underscores the importance of government initiatives aimed at establishing healthcare facilities in rural areas to reduce travel distances during emergencies. Additionally, efforts should be directed towards empowering healthcare professionals, such as doctors and nurses, to provide basic life support in remote areas. While 92% (n=322) of the population in this study identified a mode of transport, with 76% (n=266) having access to contact numbers of individuals who could assist with transportation, it's worth noting that similar studies in Rural Uganda and South Ethiopia showed lower percentages for transport identification at 65% and 24.2%, respectively, and 56.3% in Uttar Pradesh, India [11,12,18]. The increasing prevalence of individuals owning their vehicles and improvements in public transportation and road infrastructure are positive indicators. However, these findings emphasize the need for a larger percentage of the population to be prepared for transportation needs during emergencies, as access to timely transport is crucial for safe deliveries.

In India, numerous maternal and child health programs have been implemented, with notable milestones, including the reproductive and child health (RCH) phases 1 and 2. Presently, the reproductive, maternal, newborn, child, and adolescent health (RMNCH+A) program adopts a comprehensive life cycle approach to healthcare delivery. Successful incentive programs such as the Janani Suraksha Yojana (JSY) and Janani Sisu Suraksha Karyakram (JSSK) are operational across the country. Despite these initiatives, male involvement in birth preparedness remains an underemphasized area. This study sheds light upon the public health significance of promoting male partner involvement in birth preparedness and advocates for policy reforms to enhance such participation. Additionally, the study underscores the necessity for programs aimed at improving accessibility to healthcare services in rural areas, as highlighted by its findings.

Limitations

The study's participant selection was limited to villages near a tertiary care hospital, potentially introducing selection bias. Future research should aim for a more diverse and extensive geographical sampling to account for variations in healthcare facility distribution, accessibility, preferences, and community connectivity. Furthermore, a subset of study participants was employed in a hospital environment where healthcare services are often provided free or at subsidized rates for employees' families. This circumstance may have influenced the amount of money saved for emergencies and delivery, potentially compromising the generalizability of the findings. To address this limitation, future studies should encompass a more heterogeneous population across a broader geographical area. Last, it's important to note that the selected study settings were associated with urban areas and were not entirely rural, as they represented urbanized villages. Given the mixed demographic nature of the study populations, the modified Kuppuswamy scale was employed for socioeconomic classification.

## Conclusions

The findings underscore the varying levels of male involvement in birth preparedness and complication readiness. While a substantial proportion demonstrated moderate to high levels of involvement, a significant portion still exhibited low engagement in birth preparedness. Frontline workers and healthcare staff should educate men about BPCR components, allowing for informed decisions. This study's findings can help increase awareness, develop better government policies, and train healthcare staff to be more sensitive towards male participation in BPCR, ultimately reducing mortality and morbidity among pregnant women and infants.

Future research should consider a population wider than the one considered by us to provide a broader aspect. We also recommend that male partners be allowed to accompany their wives in the obstetrics OPD, USG rooms, and labor rooms, and IEC should be done regarding this. RMNCH should also add a component promoting husband involvement in the program. Various government schemes and policies should also be made to promote active male participation, like paternity leave and other benefits, etc. Capacity building for frontline workers should also be done regarding the involvement of male partners during pregnancy so that the same can be promoted at the grass-roots level. Similarly, medical practitioners and other healthcare staff could be sensitized regarding the importance of male involvement in pregnancies' information and education should be given to the couple as a unit about various physiological as well as pathological signs in the pregnancy and delivery process.

## Appendices

Scoring used in our data

We take this opportunity to share the components of BPCR and their scoring that we used in our data collection.

BPCR is a critical strategy aimed at promoting safe motherhood by encouraging pregnant individuals and their families to plan and prepare for childbirth and potential complications. It encompasses various components, including identifying the place of birth, arranging transportation, saving money for emergencies, and being aware of danger signs during pregnancy, among others. By ensuring that individuals are adequately prepared for childbirth and potential complications, BPCR contributes to reducing maternal and neonatal mortality and morbidity rates. In this context, the following table 5 presents the components of the BPCR tool and their associated scoring, which serve as essential indicators for assessing the level of preparedness among pregnant individuals and guiding interventions to improve maternal and neonatal health outcomes.

**Table 4:** Components of BPCR and their scoring

SN	Components of BPCR	Scoring
1.	Identifying the place of birth	1
2.	Identifying a birth attendant	1
3.	Arranging transport	1
4.	Saving money for emergencies	1
5.	Identifying a potential blood donor	1
6.	Knowledge of the estimated date of delivery	1
7.	Plan of taking leave during delivery/ emergencies	1
8.	Identification of a birth kit	1
9.	Discussion with the village ASHA	1
10.	Awareness of danger signs during pregnancy	1
